# A tool for analyzing and visualizing ribo-seq data at the isoform level

**DOI:** 10.1186/s12859-021-04192-7

**Published:** 2021-05-25

**Authors:** Wei-Sheng Wu, Yi-Hong Tsao, Sheng-Cian Shiue, Ting-Yu Chen, Yan-Yuan Tseng, Joseph T. Tseng

**Affiliations:** 1 Department of Electrical Engineering, National Cheng Kung University, Tainan, 701 Taiwan; 2 Department of Biotechnology and Bioindustry Sciences, National Cheng Kung University, Tainan, 701 Taiwan; 3 Center for Molecular Medicine and Genetics, School of Medicine, Wayne State University, Detroit, MI 48201 USA

**Keywords:** Ribo-seq, Ribosome profiling, Pipeline, Tool, Software, Isoform-level, Visualization

## Abstract

**Background:**

Translational regulation is one important aspect of gene expression regulation. Dysregulation of translation results in abnormal cell physiology and leads to diseases. Ribosome profiling (RP), also called ribo-seq, is a powerful experimental technique to study translational regulation. It can capture a snapshot of translation by deep sequencing of ribosome-protected mRNA fragments. Many ribosome profiling data processing tools have been developed. However, almost all tools analyze ribosome profiling data at the gene level. Since different isoforms of a gene may produce different proteins with distinct biological functions, it is advantageous to analyze ribosome profiling data at the isoform level. To meet this need, previously we developed a pipeline to analyze 610 public human ribosome profiling data at the isoform level and constructed HRPDviewer database.

**Results:**

To allow other researchers to use our pipeline as well, here we implement our pipeline as an easy-to-use software tool called RPiso. Compared to Ribomap (a widely used tool which provides isoform-level ribosome profiling analyses), our RPiso (1) estimates isoform abundance more accurately, (2) supports analyses on more species, and (3) provides a web-based viewer for interactively visualizing ribosome profiling data on the selected mRNA isoforms.

**Conclusions:**

In this study, we developed RPiso software tool (http://cosbi7.ee.ncku.edu.tw/RPiso/) to provide isoform-level ribosome profiling analyses. RPiso is very easy to install and execute. RPiso also provides a web-based viewer for interactively visualizing ribosome profiling data on the selected mRNA isoforms. We believe that RPiso is a useful tool for researchers to analyze and visualize their own ribosome profiling data at the isoform level.

**Supplementary Information:**

The online version contains supplementary material available at 10.1186/s12859-021-04192-7.

## Background

Translational regulation, which governs the mRNA translational efficiencies and protein degradation rates, is a key mechanism for protein synthesis [1, 2]. Translational regulation enables a cell to change its proteome to maintain cellular homeostasis in response to internal and external stimuli [1, 2]. Aberrations in the translational control leads to human diseases such as cancer [3]. Therefore, detailed knowledge of the molecular mechanisms of translational regulation is essential in understanding cellular homeostasis and disease.

Ribosome profiling, also called ribo-seq, is a powerful experimental technique to study translational regulation [4]. By deep sequencing of ribosome-protected mRNA fragments, ribo-seq captures a snapshot of translation at a specific time and in a specific physiological condition. Ribo-seq has been used to examine many aspects of translation such as alternative initiation, frameshift, and the dynamics of elongation and termination [5–7]. With the continuous advance of the ribo-seq technique, more biological insights into the mechanisms of eukaryotic translation will be revealed in the near future [7].

Bioinformatics tools are needed to make sense of ribo-seq data. Nowadays many software tools have been developed to analyze users’ ribo-seq data. For example, RiboProfiling [8] is a Bioconductor package that provides quality assessment and quantification of Ribo-seq data. RiboTools [9] is a Galaxy toolbox for the qualitative analysis (e.g. identification of translational ambiguities and stop codon readthrough events) on ribo-seq data. RiboGalaxy [10] provides a set of online tools (e.g. ribocount, riboplot, and riboseqR) for the analysis and visualization of ribo-seq data. Descriptions of many other tools could be found in two review papers [11, 12].

Although various existing tools have fulfilled most of the needs for ribo-seq data processing, one challenge remains to be addressed. Almost all existing tools analyze ribo-seq data at the gene level rather than the isoform level. In higher eukaryotes, many genes produce multiple mRNA isoforms [13, 14]. Different mRNA isoforms of a gene may produce a variety of proteins with distinct biological functions. Therefore, it is advantageous to analyze ribo-seq data at the isoform level so that more biological insights can be extracted from ribo-seq data.

Ribomap [15] is a widely used software tool dedicated to quantify isoform-level ribosome profiles. Ribomap assigns ribo-seq reads to different mRNA isoforms based on the estimated mRNA isoform abundance from RNA-seq. Our group previously developed a pipeline to analyze 610 human ribo-seq datasets at the isoform level and constructed HRPDviewer [16] database to provide the ribosome profiling results on each isoform in these ribo-seq datasets. To allow other researchers to do the same analyses on their ribo-seq data from a species of interest, here we implement our pipeline as an easy-to-use software tool called RPiso. RPiso is dedicated to quantify isoform-level ribosome profiles. RPiso incorporates Bowtie [17] for transcriptome mapping and RSEM [18] for isoform abundance estimation. The goals of HRPDviewer and RPiso are very different. HRPDviewer is a database which allows users to view the analyzing results of the 610 public human ribo-seq data at the isoform level. HRPDviewer does not allow users to analyze their ribo-seq data. On the contrary, RPiso is developed as an easy-to-use software tool to analyze users’ ribo-seq data. Using RPiso allows users to calculate and view the translational level of each isoform/gene from their ribo-seq data.

Compared to Ribomap, our RPiso has two unique features. First, RPiso supports analysis on multiple species. RPiso precompiled the reference mRNA transcriptomes of five species (human, mus, rat, yeast and zebrafish). On the contrary, Ribomap only precompiled the human reference mRNA transcriptome. RPiso also provides step-by-step instructions on how to prepare the reference mRNA transcriptomes of other species of interest, but Ribomap does not provide such information. Second, RPiso has a web-based viewer for interactively visualizing ribosome profiling data on the selected isoforms while Ribomap does not provide any visualization. RPiso’s online viewer helps users to find out novel biological insights. By viewing the ribo-seq data mapped on different mRNA isoforms of a gene, users can know which mRNA isoforms are highly or lowly translated in the physiological condition under study and gain an accurate understanding of differential translational regulation of different isoforms of a gene.

Here we give an example. Human cyclin G1 (encoded by gene CCNG1) plays important roles during the DNA damage response and its dysfunctions lead to cancers. CCNG1 has two mRNA isoforms (NM_004060 and NM_199246). Using RPiso to analyze ribo-seq data from a cell cycle study in Hela cell [19], we have shown in our HRPDviewer study [16] that the cyclin G1 mRNA isoform NM_004060 is constitutively translated throughout the cell cycle with peak levels at early G1 phase but the other cyclin G1 mRNA isoform NM_199246 is only translated in G1 phase, indicating that the two mRNA isoforms of the gene CCNG1 may be under different translational regulations in the cell cycle process.

## Implementation

### RPiso software workflow

**Fig. 1:**
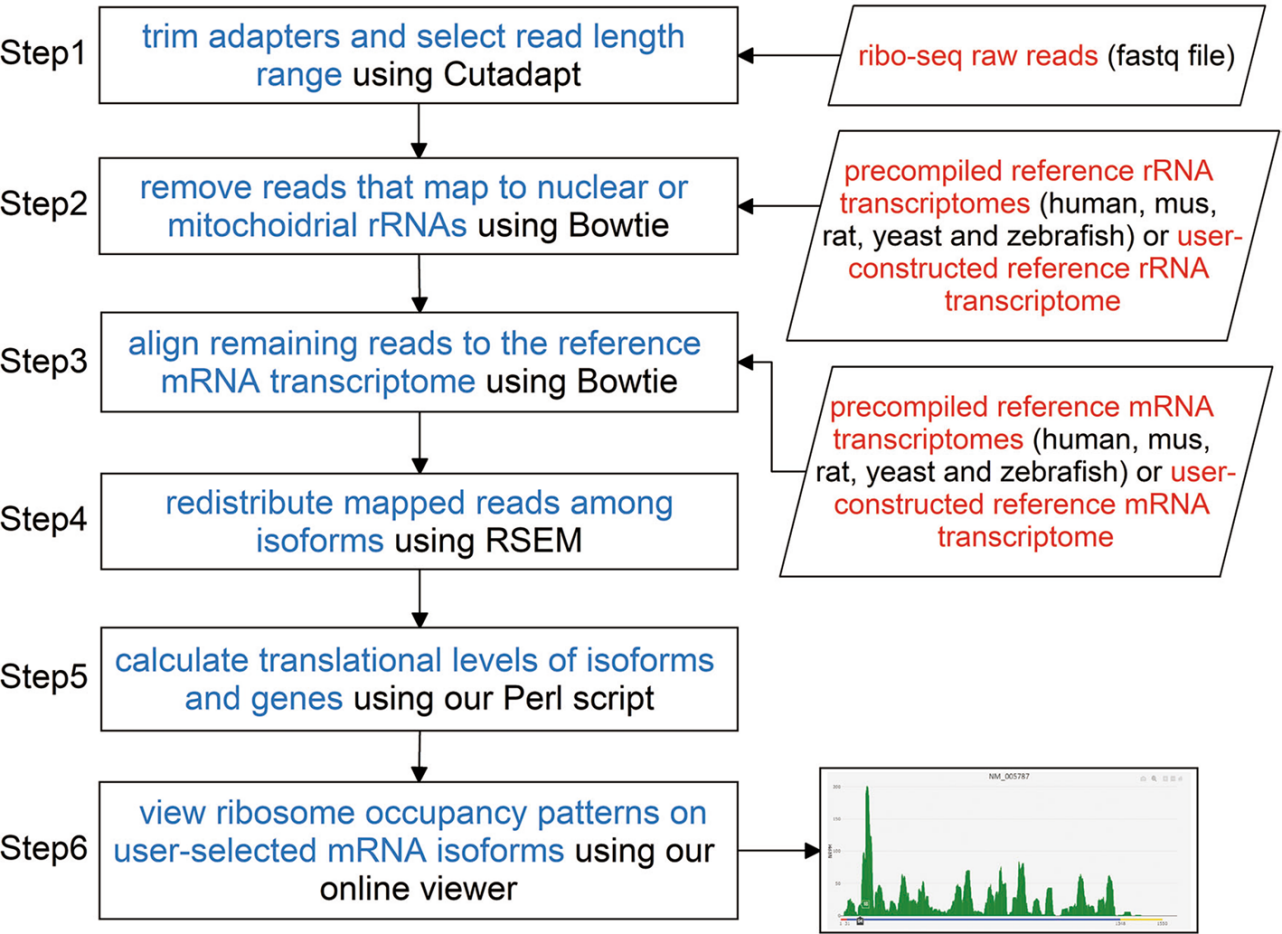
RPiso software workflow. RPiso consists of six processing steps

RPiso consists of several processing steps (Fig. 1). First, the adaptor sequences of the ribo-seq raw reads were trimmed and the reads within a certain length range (default 27 ~ 40) were kept both using Cutadapt [20]. Second, the contaminating reads (i.e. the reads mapped to the nuclear rRNAs or mitochondrial rRNAs) were removed using Bowtie [17]. The reference rRNA transcriptomes of five species (human, mus, rat, yeast, and zebrafish) were already pre-complied. Users have to construct the reference rRNA transcriptome if their ribo-seq reads come from other species. Third, the remaining reads were aligned to the reference mRNA transcriptome using Bowtie [17]. The reference mRNA transcriptomes of five species (human, mus, rat, yeast, and zebrafish) were already pre-complied. Users have to construct the reference mRNA transcriptome if their ribo-seq reads come from other species. Our software manual provides step-by-step instructions on the reference rRNA and mRNA transcriptomes preparation (Additional file 1). Fourth, the redistribution of the mapped reads among isoforms was accomplished using RSEM [18]. RSEM uses a generative statistical model which handles read mapping uncertainty in a statistically rigorous manner [18, 21]. Although RSEM was originally designed for RNA-seq, we have shown in our HRPDviewer database paper [16] that RSEM can also be used to handle read mapping uncertainty for Ribo-seq with high accuracy. Fifth, the translational levels of each mRNA isoform and each gene were calculated using our own Perl script (RPisoCalculation.pl). The translational level of an mRNA isoform is defined as the average Normalized Reads Per Kilobase per Million mapped reads (NRPKM) of its coding region. The translational level of a gene is defined as the sum of the translational levels of all its mRNA isoforms. The detailed mathematical formula could be found in our HRPDviewer database paper [16]. Finally, the ribosome occupancy patterns on the user-selected mRNA isoforms could be seen using our web-based viewer, which was developed in Python using the Django MTV framework. The ribosome occupancy patterns were plotted by a feature-rich JavaScript library called Plotly.js. If users do not want to use our web-based viewer, we also provide an.html file which contains all the figures of the ribosome occupancy patterns on the user-selected mRNA isoforms.

### Configuration of RPiso software

**Fig. 2:**
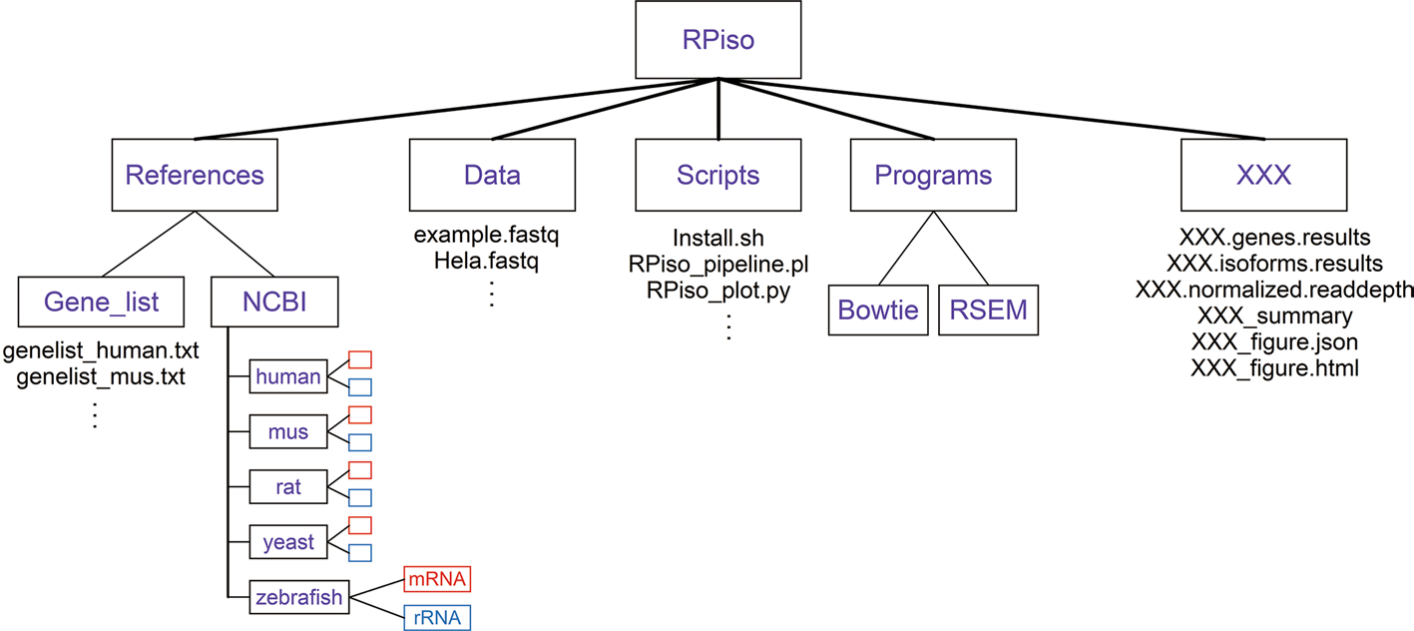
Configuration of RPiso software. The first layer is the “RPiso” directory. The second layer consists of five directories (“Data”, “References”, “Scripts”, “Programs”, and “XXX”). XXX stands for the user-defined output folder name

The configuration of RPiso is shown in Fig. 2. The first layer is the “RPiso” directory. The second layer consists of five directories (“Data”, “References”, “Scripts”, “Programs”, and “XXX”). The “Data” directory stores a user’s ribo-seq fastq file. The “References” directory contains two sub-directories. The “NCBI” subdirectory contains the reference transcriptome files for both the mRNAs and rRNAs of five species (human, mus, rat, yeast, and zebrafish) retrieved from NCBI. The “Gene_list” subdirectory contains lists of user-given gene names whose Ribo-seq profiles could be visualized by our web-based viewer. The “Scripts” directory contains all the scripts of RPiso. Users have to execute RPiso in this directory. The “Programs” directory contains two state-of-the-art read-processing tools (Bowtie-1.2.2-linux-x86_64 and RSEM-1.3.1) used in our RPiso software. The “XXX” directory contains all the output files of our RPiso software after analyzing users’ ribo-seq.fastq file. XXX stands for the user-defined output folder name. The six output files in the “XXX” directory are introduced as follows. First, the “XXX.genes.results” file contains the translational levels of all genes. Second, the “XXX.isoforms.results” file contains the translational levels of all isoforms. Third, “XXX.normalized.readdepth” file contains the normalized reads per million mapped reads (NRPM) of all the positions on each isoform. Fourth, XXX_summary file summarizes the mapping rate of each processing step of our RPiso software tool. Fifth, XXX_figure.json file contains the ribosome occupancy patterns on all isoforms of the user-selected genes (given in the “Gene_list” folder) that can be interactively visualized by our online viewer (http://cosbi7.ee.ncku.edu.tw/RPiso/). Sixth, XXX_figure.html file contains all the figures of the ribosome occupancy patterns on the user-selected mRNA isoforms. This alternative is for those users who do not want to use our online viewer.

## Results and discussion

### The usage of RPiso software

**Fig. 3:**
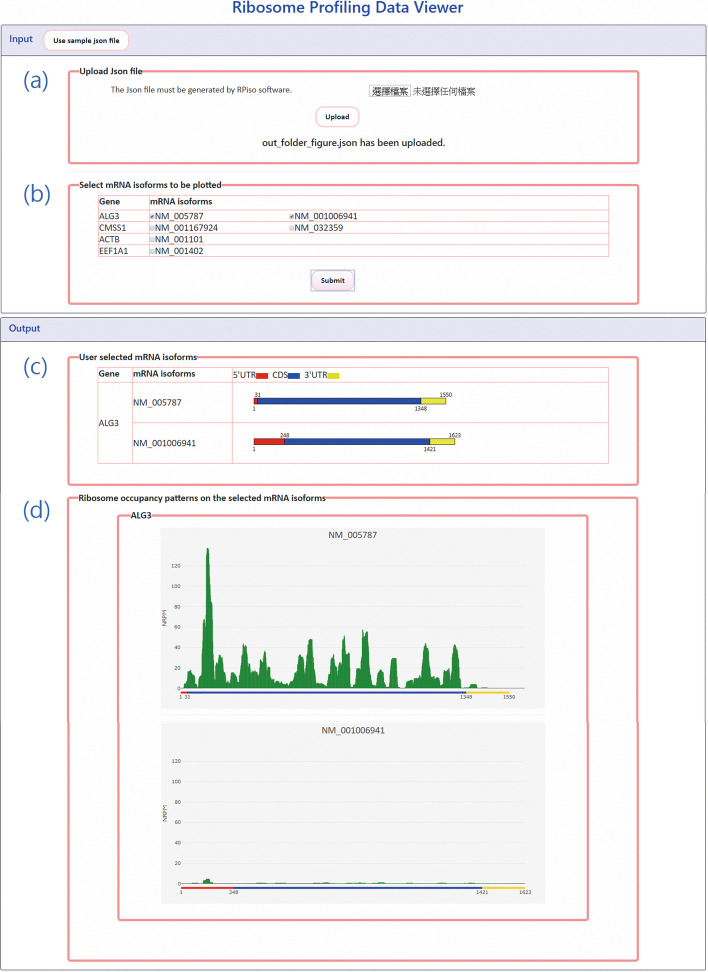
RPiso’s online viewer. To use the online viewer, users have to (**a**) upload the Json file generated by RPiso and (**b**) select the mRNA isoforms to be plotted. After submission, users will see (**c**) the information of 5’UTR, CDS, and 3’UTR for all selected mRNA isoforms and (**d**) the ribosome occupancy patterns on all the selected mRNA isoforms. The value on y-axis represents the normalized reads per million mapped reads (NRPM)

First, download RPiso.tar.gz from our website (http://cosbi7.ee.ncku.edu.tw/RPiso/). Second, decompress RPiso.tar.gz in a Linux system and users will have the following four folders: “Data”, “References”, “Scripts”, and “Programs”. Third, run Install.sh in the “Scripts” folder. This shell script will install three programs (Cutadapt 1.18, Bowtie-1.2.2-linux-x86_64 and RSEM-1.3.1) and construct the rRNA and mRNA transcriptome reference indices of five pre-compiled species (human, mus, rat, yeast, and zebrafish). Users need to do extra steps to construct the rRNA and mRNA transcriptome reference indices of the species of interest other than the five pre-complied species. The detailed instructions can be found in our RPiso manual (Additional file 1). To serve users who are familiar with Docker, we also provide a docker image of RPiso at https://hub.docker.com/r/n26091225/rpiso. Fourth, put users’ ribo-seq data in the “Data” folder. Here we use a part of the ribo-seq data of human Hela cell with RPL19 (Ribosomal Protein L19) knockdown from our lab as a sample data (named example.fastq). Fifth, run our RPiso pipeline (RPiso_pipeline.pl) in the “Scripts” folder as follows: “nohup perl RPiso_pipeline.pl -adapter CTGTAGGCACCATCAAT -species human -output ExOut example.fastq &”. The first parameter “-adapter” specifies the adapter sequence (e.g. CTGTAGGCACCATCAAT). The second parameter “-species” specifies the species being analyzed (e.g. human). The third parameter “-output” specifies the output folder name (e.g. ExOut). The final parameter specifies the user’s ribo-seq file name (e.g. example.fastq). After running RPiso_pipeline.pl, users will find an output folder (e.g. ExOut) with six files (ExOut.genes.results, ExOut.isoforms.results, ExOut.normalized.readdepth, ExOut_summary, ExOut_figure.json and ExOut_figure.html). The descriptions of these six files have been mentioned in the previous subsection “Configuration of RPiso software”. Finally, upload ExOut_figure.json into our online viewer. Users will see the ribosome occupancy patterns on all positions of all the isoforms of the user-selected genes (Fig. 3). If users do not want to use our web-based viewer, they can just open ExOut_figure.html to see all the figures of the ribosome occupancy patterns on the user-selected mRNA isoforms.

### Our RPiso estimates isoform abundance more accurately than Ribomap does

Ribomap [15] is a widely used software tool dedicated to quantify isoform-level ribosome profiles. Since our RPiso software tool also aims to provide isoform-level analysis on the ribo-seq data, the performance comparison between RPiso and Ribomap is a necessity. Following the authors of Ribomap, the human Hela cell ribo-seq data (GSM546920) in Guo et al. [22] was used as a testing dataset to evaluate the performance.

As ribo-seq footprints primarily originate from CDS regions, accurate attribution of footprints to a particular isoform over others would rely on that isoform's unique differences in CDS exonic sequence composition. (Note that this rationale has been used in HRPDviewer [16] for performance evaluation.) Assume that a gene of interest has two isoforms (isoform A and isoform B). If the unique exon of isoform A has more ribo-seq footprints than the unique exon of isoform B has, then a good isoform-level software tool should redistribute more footprints to isoform A than to isoform B. That is, the translational level of isoform A should be higher than that of isoform B. Here we used this assertion as the performance index to compare the performance of Ribomap [15] and our RPiso.

**Fig. 4:**
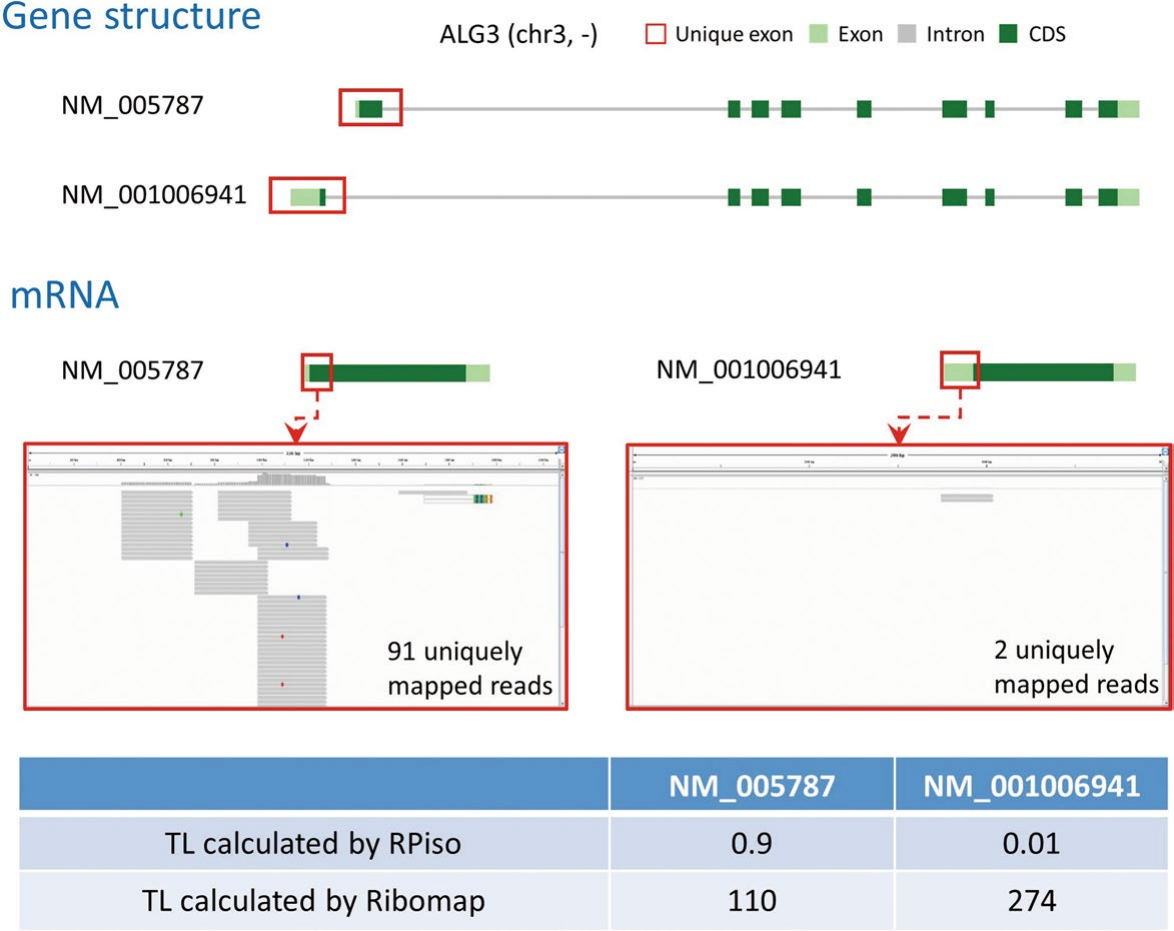
Performance comparison of RPiso and Ribomap using the gene ALG3. ALG3 has two isoforms (NM_005787 and NM_001006941). Each isoform has its unique exon. Using Integrative Genomics Viewer [23] to visualize the ribo-seq footprints (from the BAM file generated by Bowtie [17]) shows that the unique exon of NM_005787 has much more ribo-seq footprints (91 vs. 2 uniquely mapped reads) than the unique exon of NM_001006941 has. Therefore, a good isoform-level software tool should redistribute more footprints to NM_005787 than to NM_001006941. That is, the translational level (TL) of NM_005787 should be higher than that of NM_001006941. Our RPiso supports this assertion (0.9 vs. 0.01) while Ribomap contradicts this assertion (110 vs. 274). Therefore, our RPiso outperformed Ribomap in this case

To make it easy to apply the above rationale, here we only consider genes with two isoforms. From all genes in the human genome, we selected 106 human genes with two isoforms. The gene collection criteria are as follows: (1) each gene must have exactly two isoforms, (2) each isoform must have exactly one unique exon, and (3) at least one of the two unique exons must have ribo-seq footprints. By checking the outputs of both software on these 106 genes, we found that RPiso and Ribomap have the same assertions on 77 genes but have opposite assertions on the other 29 genes. Therefore, the results on these 29 genes can be used to compare the performance of RPiso and Ribomap. Take the gene ALG3 as an example. ALG3 has two isoforms (NM_005787 and NM_001006941). Each isoform has its unique exon. Using Integrative Genomics Viewer [23] to visualize the ribo-seq footprints (from the BAM file generated by Bowtie) shows that the unique exon of NM_005787 has much more ribo-seq footprints (91 vs. 2 uniquely mapped reads) than the unique exon of NM_001006941 has (Fig. 4). Therefore, the translational level of NM_005787 should be higher than that of NM_001006941. Our RPiso supports this assertion (0.9 vs. 0.01) while Ribomap contradicts this assertion (110 vs. 274). Therefore, our RPiso outperformed Ribomap in this case. In total, our RPiso outperformed Ribomap in 86% (25/29) cases (Table 1), suggesting that our RPiso estimates isoform abundance more accurately than Ribomap does.

**Table 1:** The 29 genes (with two isoforms) used to compare the performance of RPiso and Ribomap

Gene name	isoform A versus isoform B	Ribo-seq reads uniquely mapped to the unique exon of each isoform (by Bowtie): A versus B	TL(A) versus TL(B) calculated by RPiso	TL(A) versus TL(B) calculated by by Ribomap
ALG3	NM_005787 versus NM_001006941	91 versus 2 (A > B)	0.9 versus 0.01 (A > B)	110 versus 274 (A < B)
CNRIP1	NM_015463 versus NM_001111101	1 versus 0 (A > B)	0.01 versus 0 (A > B)	1 versus 2 (A < B)
COL5A1	NM_000093 versus NM_001278074	11 versus 0 (A > B)	0.69 versus 0 (A > B)	92 versus 1104 (A < B)
COQ6	NM_182476 versus NM_182480	9 versus 4 (A > B)	0.3 versus 0.11 (A > B)	12 versus 162 (A < B)
CRY2	NM_021117 versus NM_001127457	4 versus 0 (A > B)	0.06 versus 0 (A > B)	4 versus 30 (A < B)
EEF1E1	NM_001135650 versus NM_004280	124 versus 0 (A > B)	1.76 versus 0.6 (A > B)	135 versus 232 (A < B)
EGLN3	NM_022073 versus NM_001308103	30 versus 0 (A > B)	0.55 versus 0.02 (A > B)	22 versus 98 (A < B)
GALNT2	NM_004481 versus NM_001291866	181 versus 0 (A > B)	4.21 versus 0 (A > B)	234 versus 1926 (A < B)
KLF10	NM_005655 versus NM_001032282	53 versus 0 (A > B)	1.7 versus 0 (A > B)	89 versus 681 (A < B)
LCORL	NM_001166139 versus NM_153686	22 versus 13 (A > B)	0.17 versus 0.15 (A > B)	31 versus 98 (A < B)
LIG3	NM_013975 versus NM_002311	57 versus 0 (A > B)	0.4 versus 0.01 (A > B)	65 versus 300 (A < B)
LRIG3	NM_153377 versus NM_001136051	9 versus 0 (A > B)	0.09 versus 0.04 (A > B)	17 versus 128 (A < B)
LRP5	NM_002335 versus NM_001291902	3 versus 0 (A > B)	0.46 versus 0 (A > B)	321 versus 338 (A < B)
LYSMD1	NM_001136543 versus NM_212551	7 versus 0 (A > B)	0.18 versus 0 (A > B)	7 versus 28 (A < B)
MAP1S	NM_018174 versus NM_001308363	5 versus 0 (A > B)	0.15 versus 0 (A > B)	10 versus 135 (A < B)
MAT2B	NM_013283 versus NM_182796	23 versus 0 (A > B)	3.61 versus 0 (A > B)	58 versus 1068 (A < B)
NAF1	NM_138386 versus NM_001128931	127 versus 0 (A > B)	1.2 versus 0 (A > B)	133 versus 406 (A < B)
NDUFA11	NM_175614 versus NM_001193375	28 versus 0 (A > B)	1.92 versus 0 (A > B)	29 versus 355 (A < B)
NSMAF	NM_001144772 versus NM_003580	17 versus 13 (A > B)	0.11 versus 0.09 (A > B)	25 versus 167 (A < B)
NT5DC2	NM_001134231 versus NM_022908	62 versus 0 (A > B)	0.67 versus 0 (A > B)	62 versus 296 (A < B)
PPIL3	NM_130906 versus NM_032472	18 versus 0 (A > B)	0.59 versus 0 (A > B)	61 versus 75 (A < B)
PRKG1	NM_006258 versus NM_001098512	42 versus 0 (A > B)	0.2 versus 0 (A > B)	44 versus 77 (A < B)
RASA1	NM_002890 versus NM_022650	176 versus 0 (A > B)	0.45 versus 0 (A > B)	192 versus 321 (A < B)
RFC3	NM_002915 versus NM_181558	240 versus 0 (A > B)	2.01 versus 1.43 (A > B)	279 versus 808 (A < B)
RPS15	NM_001018 versus NM_001308226	22 versus 0 (A > B)	18.06 versus 0 (A > B)	24 versus 2482 (A < B)
CPD	NM_001304 versus NM_001199775	9 versus 0 (A > B)	0.11 versus 0.52 (A < B)	640 versus 33 (A > B)
PPP2R2A	NM_002717 versus NM_001177591	12 versus 0 (A > B)	0.44 versus 1.77 (A < B)	859 versus 39 (A > B)
RPL14	NM_001034996 versus NM_003973	3 versus 0 (A > B)	0.2 versus 65.57 (A < B)	12,815 versus 83 (A > B)
SUCLG2	NM_003848 versus NM_001177599	54 versus 0 (A > B)	0.41 versus 1.98 (A < B)	911 versus 16 (A > B)

Take the gene ALG3 as an example. ALG3 has two isoforms (NM_005787 and NM_001006941). Each isoform has its unique exon. The unique exon of NM_005787 has much more Ribo-seq footprints (91 vs. 2 uniquely mapped reads) than the unique exon of NM_001006941 has. Therefore, the translational level (TL) of NM_005787 should be higher than that of NM_001006941. Our RPiso supports this assertion (0.9 vs. 0.01) while Ribomap contradicts this assertion (110 vs. 274). Therefore, our RPiso outperformed Ribomap in this case. In total, our RPiso outperformed Ribomap in 86% (25/29) of the cases (bold-faced names), suggesting that our RPiso estimates isoform abundance more accurately than Ribomap does

## Conclusions

In this study, we developed RPiso software tool to provide isoform-level ribosome profiling analyses. RPiso is very easy to install and execute. Compared to Ribomap (a widely used software tool which provides isoform-level ribosome profiling analyses), our RPiso has four advantages. First, while Ribomap needs RNA-seq to assign ribo-seq reads to different mRNA isoforms, our RPiso can do the same task only based on ribo-seq reads alone. That is, Ribomap needs both RNA-seq and ribo-seq as inputs while RPiso can analyze ribo-seq alone. Second, RPiso estimates isoform abundance more accurately than Ribomap does. RPiso outperforms Ribomap in 86% (25/29) of the case studies (Table 1). Third, RPiso supports analysis on multiple species while Ribomap supports only human. RPiso precompiled the reference mRNA transcriptomes of five species (human, mus, rat, yeast, and zebrafish). For each species, the following data are provided: rRNA sequences, mRNA sequences, mRNA annotations (mRNA length, 5’UTR length, CDS length, and 3’UTR length, and mRNA-gene mapping). From mRNA-gene mapping information, users can know all the mRNA isoforms of a gene of interest. Moreover, we have run Bowtie to generate the rRNA and mRNA transcriptome reference indices of each of the five species. Therefore, if the user’s ribo-seq data belong to these five species, users then can run RPiso without doing extra steps to construct the transcriptome reference indices first. RPiso also provides step-by-step instructions on how to prepare the reference mRNA transcriptomes on other species of interest, but Ribomap does not provide such information. Fourth, RPiso has a web-based viewer for interactively visualizing ribosome occupancy patterns on the selected isoforms while Ribomap does not give any visualization. RPiso’s online viewer helps users to find out novel biological insights. For example, by viewing the ribo-seq data mapped on two isoforms (NM_005787 and NM_001006941) of ALG3, users can know the translational level of NM_005787 is much higher than that of NM_001006941 (Fig. 3d) in the physiological condition under study (i.e. human Hela cell with RPL19 (Ribosomal Protein L19) knockdown), indicating that the two isoforms of ALG3 may be under different translational regulation. We believe that RPiso is a useful software tool for researchers to analyze and visualize their own ribo-seq data at the isoform level.

## Availability and requirements

Project name: RPiso.

Project home page: [main site] http://cosbi7.ee.ncku.edu.tw/RPiso/, [backup site 1] http://cosbi4.ee.ncku.edu.tw/RPiso/, and [backup site 2] http://cosbi6.ee.ncku.edu.tw/RPiso/.

Operating system(s): Linux ubuntu 14.04 LTS (or 16.04 LTS).

Programming language: Perl 5.22.1 and Python 2.7.12 (or 3.5.2).

Other requirements: Cutadapt 1.18, Bowtie-1.2.2-linux-x86_64 and RSEM-1.3.1.

License: none required.

Any restrictions to use by non-academics: no restriction.

## Supplementary Information

**Additional file 1**: RPiso manual.

## Data Availability

All the data in RPiso are available at http://cosbi7.ee.ncku.edu.tw/RPiso/.

## Abbreviations

NRPKM: Normalized Reads Per Kilobase per Million mapped reads
NRPM: Normalized Reads Per Million mapped reads
RP: Ribosome profiling
