# Supplementary material for: A tool for analyzing and visualizing ribo-seq data at the isoform level
Source: BMC Bioinformatics. 2021 May 25;22(Suppl 10):271. doi: 10.1186/s12859-021-04192-7 (PMC8323483; doi:10.1186/s12859-021-04192-7)
Supplement: Supplementary file 1 — Additional file 1: RPiso manual. [file 12859_2021_4192_MOESM1_ESM.pdf]

Contents of RPiso manual

1. **RPiso software workflow --- p.2**
2. **Configuration of RPiso software --------------------------------- p.4**
3. **The usage of RPiso software ------------------------------------- p.7**
4. **Prepare the reference transcriptome of your species --- p.11**
5. **Run RPiso for the ribo-seq data from your species ----- p.13**
6. **References --- p.15**

RPiso software workflow

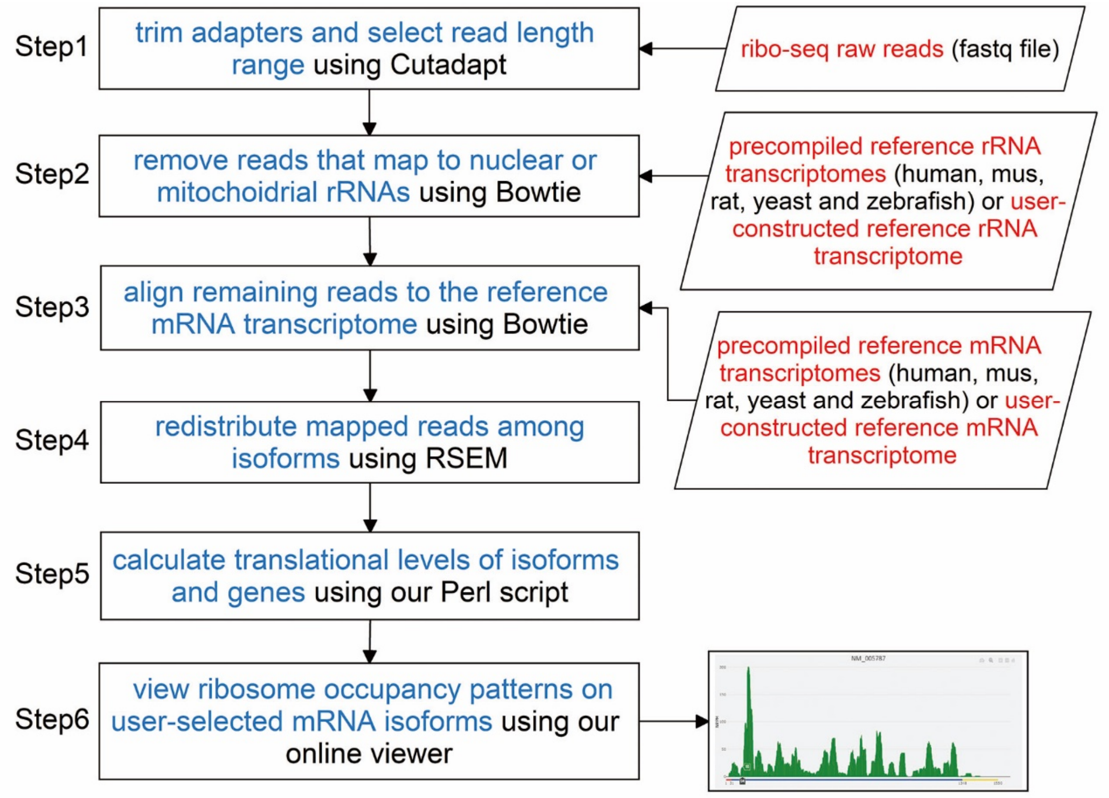

Figure 1 - RPiso software workflow. RPiso consists of six processing steps.

RPiso consists of six processing steps.

1. The adaptor sequences of the ribo-seq raw reads were trimmed and the reads within a certain length range (default 27~40) were kept both using Cutadapt [1].
2. The contaminating reads (i.e. the reads mapped to the nuclear rRNAs or mitochondrial rRNAs) were removed using Bowtie [2]. The reference rRNA transcriptomes of five species (human, mus, rat, yeast, and zebrafish) were already pre-complied. Users have to construct the reference rRNA transcriptome if their ribo-seq reads come from other species (**See p.11 for the details**).
3. The remaining reads were aligned to the reference mRNA transcriptome using Bowtie. The reference mRNA transcriptomes of five species (human, mus, rat, yeast, and zebrafish) were already pre-complied. Users have to construct the reference mRNA transcriptome if their ribo-seq reads come from other species (**See p.11 for the details**).

4. The redistribution of the mapped reads among isoforms was accomplished using RSEM [3]. RSEM uses a generative statistical model which handles read mapping uncertainty in a statistically rigorous manner [3,4]. Although RSEM was originally designed for RNA-seq, we have shown in our HRPDviewer database paper [5] that RSEM can also be used to handle read mapping uncertainty for Ribo-seq with high accuracy.
5. The translational levels of each mRNA isoform and each gene were calculated using our own Perl script (RPisoCalculation.pl).
   (a) **The translational level (TL) of an mRNA isoform** is defined as the average Normalized Reads Per Kilobase per Million mapped reads (NRPKM) of its coding region as the following formula

$$TL_{mRNA} = \frac{\sum_{i=1}^{L} NRPM_i}{L/_{1000}}$$

   where ***NRPM*** stands for Normalized Reads Per Million mapped reads, ***L*** is the length (in bps) of the coding region and ***i*** is the *i*-th position of the coding region. The more details of the mathematical formula could be found in our HRPDviewer database paper [5].
   (b) **The translational level of a gene** is defined as the sum of the translational levels of all its mRNA isoforms.
6. The ribosome occupancy patterns on the user-selected mRNA isoforms could be seen using our web-based viewer, which was developed in Python using the Django MTV framework. The ribosome occupancy patterns were plotted by a feature-rich JavaScript library called Plotly.js.

Configuration of RPiso software

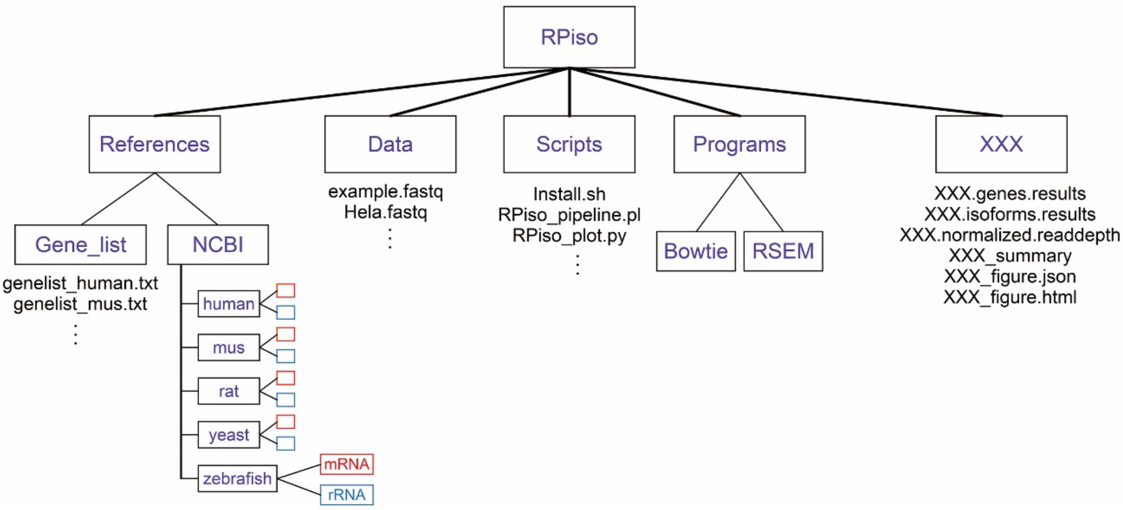

Figure 2 - Configuration of RPiso software.

The first layer is the "RPiso" directory.

The second layer consists of five directories ("Data", "References", "Scripts", "Programs", and "XXX"):

1. The "Data" directory stores a user's ribo-seq fastq file.
2. The "References" directory contains two sub-directories. The "NCBI" subdirectory contains the reference transcriptome files for both the mRNAs and rRNAs of five species (human, mus, rat, yeast, and zebrafish) retrieved from NCBI. The "Gene_list" subdirectory contains lists of user-given gene names whose Ribo-seq profiles could be visualized by our web-based viewer.
3. The "Scripts" directory contains all the scripts of RPiso. Users have to execute RPiso in this directory.
4. The "Programs" directory contains two state-of-the-art read-processing tools (Bowtie-1.2.2-linux-x86_64 and RSEM-1.3.1) used in our RPiso software.
5. The "XXX" directory contains all the output files of our RPiso software after analyzing users' ribo-seq fastq file. XXX stands for the user-defined output folder name. The output files in the "XXX" directory are as follows:

(a) The “XXX.genes.results” file contains the translational levels of all genes.

```
Gene     Transcript_ID   Expression
AAMP     NM_001087,NM_001302545  4.65
AANAT    NM_001166579,NM_001088  0
AAR2     NM_001271874,NM_015511  1.31
AARD     NM_001025357     0
AARS     NM_001605        1.72
AARS2    NM_020745        0.2
AARSD1   NM_001261434     0.9
```

(b) The “XXX.isoforms.results” file contains the translational levels of all isoforms.

```
Transcript_ID   Gene     Expression
NM_001318038    A4GALT   0.00
NM_017436       A4GALT   0.53
NM_001173466    AAAS     0.79
NM_015665       AAAS     0.74
NM_001319839    AACS     0.00
NM_001319840    AACS     0.00
NM_023928       AACS     0.56
```

(c) The “XXX.normalized.readdepth” file contains the length and normalized reads per million mapped reads (NRPM) of all the positions on each isoform.

```
NM_001135650    609     0 0 0 0 0 0 0 0 0 0 0 0 0 0 0 0 0 0 0 0 0 0 0 0 0 0 0 0 0 0 0 0 0 0 0 0 0 0 0
0 0 0 0 0 0 0 0 0 0 0 0 0 0 0 0 0 0 0 0 0 0 0 0 0 0 0 0 0 0 0 0 0 0 0 0 0 0 0 0 0 0 0 0 0 0 0 0 0 0 0
0 0 0 0 0 0 0.773440454755372 0.773440454755372 0.773440454755372 1.46482093613782 1.46482093613782 1.
46482093613782 1.46482093613782 1.5055312405786 1.5055312405786 1.5055312405786 1.5055312405786 1.5055
312405786 1.5055312405786 1.5055312405786 1.5055312405786 1.5055312405786 1.5055312405786 1.5055312405
786 1.5055312405786 2.0338922198686 2.0338922198686 2.0338922198686 2.0338922198686 2.0338922198686 2.
0338922198686 2.0338922198686 2.0338922198686 2.0338922198686 2.0338922198686 0.854018823874264 0.8540
18823874264 0.854018823874264 0.569067595089484 0.569067595089484 0.569067595089484 0.569067595089484
0.976141130366912 0.976141130366912 0.976141130366912 0.976141130366912 0.976141130366912 0.9761411303
66912 0.976141130366912 0.976141130366912 0.976141130366912 0.44778138062401 0.44778138062401 0.447781
38062401 0.44778138062401 0.44778138062401 0.44778138062401 0.44778138062401 0.44778138062401 0.447781
38062401 0.44778138062401 0.44778138062401 0.44778138062401 0.44778138062401 0.44778138062401 0.447781
38062401 0.44778138062401 0.44778138062401 0.44778138062401 0.44778138062401 0.44778138062401 0 0 0 0
0 0 0 0 0 0 0 0 0 0 0 0 0 0 0 0 0 0 0 0 0 0 0 0 0 0 0 0 0 0 0.243986408094551 0.243986408094551 0.2439
86408094551 0.243986408094551 0.936746441321832 0.936746441321832 0.936746441321832 0.936746441321832
0.936746441321832 0.936746441321832 0.936746441321832 0.936746441321832 0.936746441321832 1.1400692677
6536 1.66982332391828 1.66982332391828 1.66982332391828 1.66982332391828 1.66982332391828 1.6698233239
1828 1.66982332391828 1.66982332391828 1.66982332391828 1.66982332391828 1.66982332391828 1.6698233239
```

(d) The “XXX _summary” file summarizes the mapping rate of each processing step of our RPiso software tool.

```
Sample name      ExOut
Raw Read         5057686
With adapters    4938465 (97.64%)
Too short        214436 (4.24%)
Too long         127823 (2.53%)
Passing filters  4715427 (93.23%)

The percentage is compared with passing cutadapt filters
Mitochondria rRNAs       1740 (0.04%)
human rRNAs      1685472 (35.74%)
Coding transcripts       1461727 (31.00%)
Unmapped to transcriptome        1566488 (33.22%)
```

(e) “XXX_figure.json” file contains the ribosome occupancy patterns on all isoforms of the user-selected genes (given in the “Gene_list” folder) that can be visualized by our web-based viewer.

(f) “XXX_figure.html” file contains all the figures of the ribosome occupancy patterns on all isoforms of the user-selected genes (given in the “Gene_list” folder). This alternative is for those users who do not want to use our online viewer.

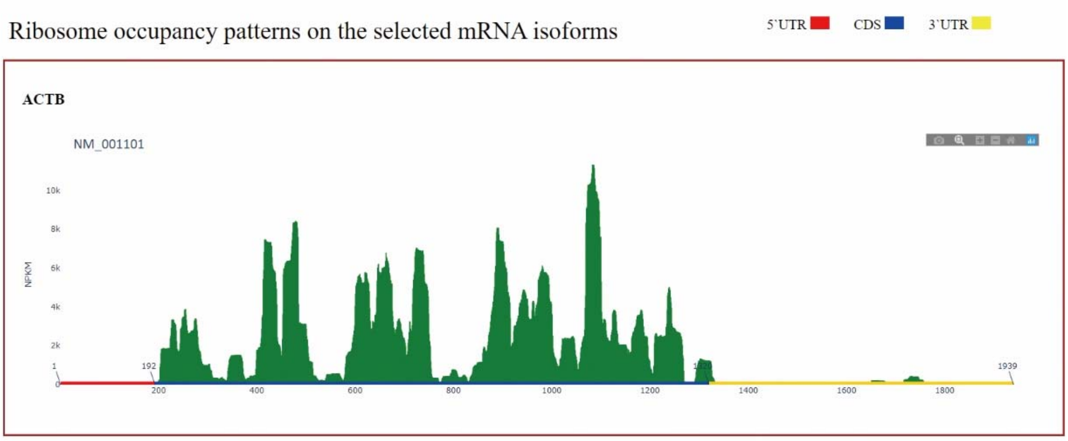

The usage of RPiso software

1. Download RPiso.tar.gz from our website (http://cosbi6.ee.ncku.edu.tw/RPiso/).
2. Decompress RPiso.tar.gz **in a Linux system** and users will have the "RPiso" folder with four subfolders: "Data", "References", "Scripts", and "Programs".
3. **Run Install.sh in the "Scripts" folder**. This shell script will install three programs (**Cutadapt 1.18**, **Bowtie-1.2.2-linux-x86_64** and **RSEM-1.3.1**) and construct the rRNA & mRNA transcriptome reference indices of five pre-compiled species (human, mus, rat, yeast, and zebrafish). Users need to do extra steps to construct the rRNA & mRNA transcriptome reference indices of the species of interest other than the five pre-complied species (**see p.11 for details**).
4. **Put users' ribo-seq data in the "Data" folder**. Here we use a part of the ribo-seq data of human Hela cell with RPL19 (Ribosomal Protein L19) knockdown from our lab as a sample data (named example.fastq).
5. **In the "Gene_list" subfolder inside the "References" folder**, prepare a file named genelist_*[species name]*.txt (e.g. genelist_human.txt). This file should contain the user-given gene names whose Ribo-seq profiles could be visualized by our web-based viewer.
6. **Run our RPiso software (RPiso_pipeline.pl) in the "Scripts" folder** as follows:

```
nohup perl RPiso_pipeline.pl \
  -adapter CTGTAGGCACCATCAAT \
  -species human \
  -output ExOut \
 example.fastq &
```

   (a) The parameter "-adapter" specifies the adapter sequence (e.g. CTGTAGGCACCATCAAT).
   (b) The parameter "-species" specifies the species being analyzed (e.g. human).
   (c) The parameter "-output" specifies the output folder name (e.g. ExOut).
   (d) The last parameter specifies the user's ribo-seq file name (e.g. example.fastq).

   More parameters which can be specified are introduced as follows.

Parameter	Setting	Explanation
-contamination	0,1,2	0: do not remove any contaminating reads<br>1: remove reads mapped to mitochondria RNAs<br>2: remove reads mapped to nuclear RNAs (Default: 1,2)
-min	<int>	Discard reads shorter than <int> when running Cutadapt (Default: 27)
-max	<int>	Discard reads longer than <int> when running in Cutadapt (Default: 40)
-p	<int>	Number of threads used by Bowtie (Default: 1)
-seedlen	<int>	Seed length used by Bowtie (Default: 23)
-seed_mismatch	0-3	max # of mismatches in the seed when running Bowtie (Default: 2)

7. After running RPiso_pipeline.pl, users will find **an output folder (e.g. ExOut)** with six files:
   (a) ExOut.genes.results
   (b) ExOut.isoforms.results
   (c) ExOut.normalized.readdepth
   (d) ExOut_summary
   (e) ExOut_figure.json
   (f) ExOut_figure.html

8. Upload ExOut_figure.json into our web-based viewer (http://cosbi6.ee.ncku.edu.tw/RPiso/). Users will see the ribosome occupancy patterns on all positions of all the isoforms of the user-selected genes.

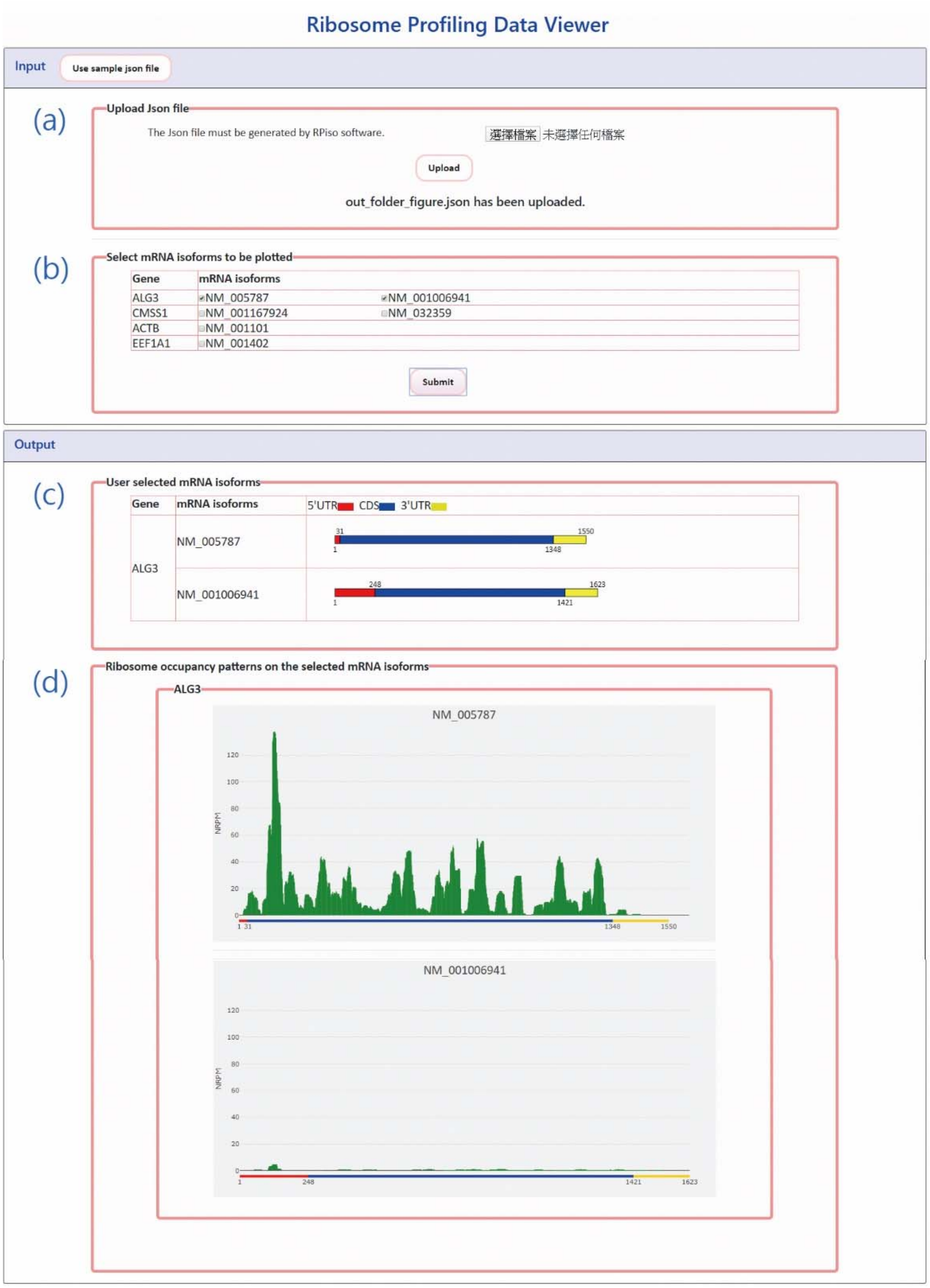

Figure 3 - RPiso's online viewer. To use the online viewer, users have to (a) upload the Json file generated by RPiso and (b) select the mRNA isoforms to be plotted. After submission, users will see (c) the information of 5'UTR, CDS, and 3'UTR for all selected mRNA isoforms and (d) the ribosome occupancy patterns on all the selected mRNA isoforms. The value on y-axis represents the normalized reads per million mapped reads (NRPM).

If users do not want to use our web-based viewer, they can just open ExOut_figure.html to see the ribosome occupancy patterns on all positions of all the isoforms of the user-selected genes.

After running RPiso_pipeline.pl for the first time, if users want to see the ribosome occupancy patterns on the isoforms of another set of genes, they do not need to rerun RPiso_pipeline again. They only need to do the followings.

(i) Replace the old gene names with the new gene names in the **genelist_human.txt** file (located at /RPiso/**References/Gene_list**/genelist_human.txt).

(ii) **Run our RPiso_plot.py in the ”Scripts” folder** as follows:

```
python RPiso_plot.py \
-readdepth ../ExOut/ExOut.normalized.readdepth\
-genelist ../References/Gene_list/genelist_human.txt \
-coord ../References/NCBI/human/mRNA/human_NM.coord
```

In the “ExOut” folder, users will see two updated files: **ExOut_figure.json** and **ExOut_figure.html**. Both files contains the ribosome occupancy patterns on the isoforms of the newly selected genes.

Prepare the reference transcriptome of your species of interest (e.g. YYY)

1. Create a folder ("YYY") in the NCBI folder and two folders ("rRNA" and "mRNA") in the "YYY" folder.

```
(python3_6_env) t50504@cosbi2:~/Bioinfo#winter/dr/RPiso/References/NCBI$ ls
human  mus  rat  yeast  YYY  zebrafish
(python3_6_env) t50504@cosbi2:~/Bioinfo#winter/dr/RPiso/References/NCBI/YYY$ ls
mRNA  rRNA
```

2. **In the "rRNA" folder**, prepare a fasta file (rRNA.fasta) containing all nucleus rRNA sequences of the species YYY.

```
>NR_003285.3 Homo sapiens RNA, 5.8S ribosomal N5 (RNA5-8SN5), ribosomal RNA
CGACTCTTAGCGGTGGATCACTCGGCTCGTGCGTCGATGAAGAACGCAGCTAGCTGCGAGAATTAATGTGAATTGCAGGACACATTGATCATCGACACTTCGAACGCACTTGC
GGCCCCGGGTTCCTCCCGGGGCTACGCCTGTCTGAGCGTCGCTT
>NR_003286.4 Homo sapiens RNA, 18S ribosomal N5 (RNA18SN5), ribosomal RNA
TACCTGGTTGATCCTGCCAGTAGCATATGCTTGTCTCAAAGATTAAGCCATGCATGTCTGAGTACGCACGGCCGGTACAGTGAAACTGCGAATGGCTCATTAAATCAGTTATG
GTTCCTTTGGTCGCTCGCTCCTCTCCTACTTGGATAACTGTGGTAATTCTAGAGCTAATACATGCCGACGGGCGCTGACCCCCTTCGCGGGGGGGATGCGTGCATTTATCAGA
```

3. **In the "rRNA" folder**, construct the reference nucleus rRNA transcriptome using the following command:

../../../../Programs/bowtie-1.2.2-linux-x86_64/bowtie-build \
-f rRNA.fasta \
rRNA

Input file: rRNA.fasta

Output files:

```
rRNA.1.ebwt  rRNA.2.ebwt  rRNA.3.ebwt  rRNA.4.ebwt  rRNA.rev.1.ebwt  rRNA.rev.2.ebwt
```

4. **In the "rRNA" folder**, prepare a fasta file (MTRNR.fasta) containing all mitochondria rRNA sequences of the species YYY.

```
>NR_137294.1 Homo sapiens mitochondrially encoded 12S ribosomal RNA (RNR1), ribosomal RNA
AATAGGTTTGGTCCTAGCCTTTCTATTAGCTCTTAGTAAGATTACACATGCAAGCATCCCCGTTCCAGTGAGTTCACCCTCTAAATCACCACGATCAAAAGGAACAAGCATCA
AGCACGCAGCAATGCAGCTCAAAACGCTTAGCCTAGCCACACCCCCACGGGAAACAGCAGTGATTAACCTTTAGCAATAAACGAAAGTTTAACTAAGCTATACTAACCCCAGG
GTTGGTCAATTTCGTGCCAGCCACCGCGGTCACACGATTAACCCAAGTCAATAGAAGCCGGCGTAAAGAGTGTTTTAGATCACCCCCTCCCCAATAAAGCTAAAACTCACCTG
AGTTGTAAAAAACTCCAGTTGACACAAAATAGACTACGAAAGTGGCTTTAACATATCTGAACACACAATAGCTAAGACCCAAACTGGGATTAGATACCCCACTATGCTTAGCC
CTAAACCTCAACAGTTAAATCAACAAAACTGCTCGCCAGAACACTACGAGCCACAGCTTAAAACTCAAAGGACCTGGCGGTGCTTCATATCCCTCTAGAGGAGCCTGTTCTGT
AATCGATAAACCCCGATCAACCTCACCACCTCTTGCTCAGCCTATATACCGCCATCTTCAGCAAACCCTGATGAAGGCTACAAAGTAAGCGCAAGTACCCACGTAAAGACGTT
AGGTCAAGGTGTAGCCCATGAGGTGGCAAGAAATGGGCTACATTTTCTACCCCAGAAAACTACGATAGCCCTTATGAAACTTAAGGGTCGAAGGTGGATTTAGCAGTAAACTA
AGAGTAGAGTGCTTAGTTGAACAGGGCCCTGAAGCGCGTACACACCGCCCGTCACCCTCCTCAAGTATACTTCAAAGGACATTTAACTAAAACCCCTACGCATTTATATAGAG
GAGACAAGTCGTAACATGGTAAGTGTACTGGAAAGTGCACTTGGACGAAC
>NR_137295.1 Homo sapiens mitochondrially encoded 16S ribosomal RNA (RNR2), ribosomal RNA
GCTAAACCTAGCCCCAAACCCACTCCACCTTACTACCAGACAACCTTAGCCAAACCATTTACCCAAATAAAGTATAGGCGATAGAAATTGAAACCTGGCGCAATAGATATAGT
ACCGCAAGGGAAAGATGAAAAATTATAACCAAGCATAATATAGCAAGGACTAACCCCTATACCTTCTGCATAATGAATTAACTAGAAATAACTTTGCAAGGAGAGCCAAAGCT
```

5. **In the "rRNA" folder**, construct the reference mitochondria rRNA transcriptome using the following command:

../../../../Programs/bowtie-1.2.2-linux-x86_64/bowtie-build \
-f MTRNR.fasta \
MTRNR

Input file: MTRNR.fasta

Output files:

```
MTRNR.1.ebwt  MTRNR.2.ebwt  MTRNR.3.ebwt  MTRNR.4.ebwt  MTRNR.rev.1.ebwt  MTRNR.rev.2.ebwt
```

6. **In the “mRNA” folder**, prepare a fasta file (YYY_NM.fa) containing all coding transcripts of the species YYY.

```
>NM_001001503
GGCTGTTCAGGCGCGGGTGCGCGCGCAGCTGCTGTGGCCCTGCTTGGTGCGCCCGCTGTCACCGCCATGGCTGCCCCGTGTTTGCTGCGGCAAGGACGAGCCGGGGCGCTGAA
GACTATGCTCCAGGAAGCCCAGGTGTTTCGAGGACTTGCTTCTACGGTTTCTTTGTCTGCGGAATCAGGGAAGAGTGAAAAGGGTCAGCCACAGAATTCCAAGAAGCAAAGTC
CACCAAAAAAGCCAGCCCCAGTGCCTGCTGAGCCGTTTGACAACACTACCTACAAGAACCTGCAGCATCATGACTACAGCACGTACACCTTCTTAGACCTCAACCTCGAACTC
TCAAAATTCAGGATGCCTCAGCCCTCCTCAGGCCGGGAGTCACCTCGACACTGAGGGCCCTCGGTGTGAAGATGAACCTTCCACCGTCTTCACTGCATCCTGGAGTGCAAAAA
TAAAATCCACTCAAGAGTCACAAGGCCCGCTGTGCATAATCGGTTTCACTTTTACCTTTTTTTTTTTTTTTTTTTTTTTTGAGACAGGGTCTCACTCTGTCACCCAGGCTGGAG
TGCAGTGGCACATTCTCGGCTCACTGCAACTTCCGCCTCCTGGGTTCAAGTGATTCTCCCACCTCAGCCTCCCAAGTAGGTGGGATTACAGGTACTCACCACCAGGTCCAGCT
AACTTTTGTATTTTTAGTAGAGACAGGGTTTCACCATGTTGGCCAGGCTGGTCTCGAACTCCTGACCTCAGATGGTCTGCCCACCTCCGCCTCCCAAAGTGCTGGGATTACAG
GCGTGAGCCACTGCGCCCGGCCACTTTCACACTTTTTACAGTGAGTGGTGAATTAGCAACAGTAACACTGATTATCCAACATATATTTTGGAATATCTACTATGTGCAAGGAA
TTTTTCTTAAACTCTAAGGTTATGAATCACTGGGCAAATCCATATAATTAGAGAATTTTAAGTGCTTTAGAGCGGTGTGATTCTACTGTGCTCAGCCTAGTCAATTTCGCATT
AAACTGATTATCAGCTGAAAAAAAAAAAAAAAAAAAAAA
>NM_001313966
ACGCACAGCCCCCCTGGGGGCCGGGGGCGGGGCCAGGCTATAAACCGCCGGTTAGGGGCCGCCATCCCCTCAGAGCGTCGGGATATCGGGTGGCGGCTCGGGACGGAGGACGC
GCTAGTGTTCTTCTGTGTGGCAGTTCAGAATGATGGATCAAGCTAGATCAGCATTCTCTAACTTGGCTGCTGAATGAAAATTCATATGTCCCTCGTGAGGCTGGATCTCAAAA
AGATGAAAATCTTGCGTTGTATGTTGAAAATCAATTTCGTGAATTTAAACTCAGCAAAGTCTGGCGTGATCAACATTTTGTTAAGATTCAGGTCAAAGACAGCGCTCAAAACT
```

7. In the “mRNA” folder, prepare a file (GeneIsoform_NM.txt) containing the following information of all coding transcripts: Gene ID and Transcript ID.

```
A1BG    NM_130786
A1CF    NM_138933
A1CF    NM_001198818
A1CF    NM_014576
A1CF    NM_001198819
A1CF    NM_138932
A1CF    NM_001198820
A2M     NM_001347423
A2M     NM_000014
```

8. **In the “mRNA” folder**, construct the reference mRNA transcriptome using the following command:

```
../../../../Programs/RSEM-1.3.1/rsem-prepare-reference \
-p 15 \
--bowtie-path ../../../../Programs/bowtie-1.2.2-linux-x86_64 \
--bowtie \
--transcript-to-gene-map GeneIsoform_NM.txt \
YYY_NM.fa \
ncbi_NM
```

Input files: GeneIsoform_NM.txt, YYY_NM.fa

Output files:

```
ncbi_NM.1.ebwt   ncbi_NM.n2g.idx.fa
ncbi_NM.2.ebwt   ncbi_NM.rev.1.ebwt
ncbi_NM.3.ebwt   ncbi_NM.rev.2.ebwt
ncbi_NM.4.ebwt   ncbi_NM.seq
ncbi_NM.grp      ncbi_NM.ti
ncbi_NM.idx.fa   ncbi_NM.transcripts.fa
```

9. **In the “mRNA” folder**, prepare a file (YYY_NM.coord) containing the following information of all coding transcripts: Transcript ID, Gene ID, RNA length, and CDS coordinates.

```
Transcript_ID   Gene      RNA    CDS
NM_001127200    GAGE2E    579    117~467
NM_001187       BAGE      1004   201~332
NM_001348289    OR10AC1   1144   101~1078
NM_001348266    OR4K3     1548   271~1248
NM_001474       GAGE4     528    83~436
NM_012149       DUX5      594    1~594
NM_021123       GAGE7     524    80~433
NM_181704       BAGE4     1840   189~308
NM_182481       BAGE3     1891   209~538
```

Run RPiso for the ribo-seq data from your species

1. **In the “Gene_list” folder** (/RPiso/References/Gene_list/), prepare a file named genelist_YYY.txt.

```
t50504@cosbi7:~/RPiso/References/Gene_list$ ls
genelist_YYY.txt  genelist_human.txt  genelist_mus.txt  genelist_rat.txt  genelist_yeast.txt  genelist_zebrafish.txt
```

   The genelist_YYY.txt file contains the gene names whose Ribo-seq profiles could be visualized by our web-based viewer.

```
ALG3
CMSS1
ACTB
EEF1A1
```

2. **Run our RPiso software (RPiso_pipeline.pl) in the ”Scripts” folder** as follows:

```
nohup perl RPiso_pipeline.pl \
   -adapter your_adapter_sequence \
   -species YYY \
   -output OutFolder \
 your_data.fastq &
```

3. After running RPiso_pipeline.pl, users will find **an output folder (e.g. OutFolder)** with six files:
   (a) OutFolder.genes.results
   (b) OutFolder.isoforms.results
   (c) OutFolder.normalized.readdepth
   (d) OutFolder_summary
   (e) OutFolder_figure.json
   (f) OutFolder_figure.html

4. Upload OutFolder_figure.json into our web-based viewer (http://cosbi6.ee.ncku.edu.tw/RPiso/). Users will see the ribosome occupancy patterns on all positions of all the isoforms of the user-selected genes. If users do not want to use our web-based viewer, they can just open OutFolder_figure.html to see all the figures.

After running RPiso_pipeline.pl for the first time, if users want to see the ribosome occupancy patterns on the isoforms of another set of genes, they do not need to rerun RPiso_pipeline again. They only need to do the followings.

(i) Replace the old gene names with the new gene names in the **genelist_YYY.txt** file (located at /RPiso/References/Gene_list/genelist_YYY.txt).

(ii) **Run our RPiso_plot.py in the "Scripts" folder** as follows:

```
python RPiso_plot.py \
-readdepth ../OutFolder/OutFolder.normalized.readdepth\
-genelist ../References/Gene_list/genelist_YYY.txt \
-coord ../References/NCBI/YYY/mRNA/YYY_NM.coord
```

In the "OutFolder" folder, users will see two updated files: **OutFolder_figure.json** and **OutFolder_figure.html**. Both files contains the ribosome occupancy patterns on the isoforms of the newly selected genes.

References

1. Martin,M. (2011) Cutadapt removes adapter sequences from high-throughput sequencing reads. *EMBnet Journal*, **17(1)**, 10.

2. Langmead,B., Trapnell,C., Pop,M. *et al.* (2009) Ultrafast and memory-efficient alignment of short DNA sequences to the human genome. *Genome Biol.*, **10(3)**, R25.

3. Li,B. and Dewey,C.N. (2011) RSEM: accurate transcript quantification from RNA-Seq data with or without a reference genome. *BMC Bioinformatics*, **12**, 323.

4. Li,B., Ruotti,V., Stewart,R.M. *et al.* (2010) RNA-Seq gene expression estimation with read mapping uncertainty. *Bioinformatics*, **26(4)**, 493-500.

5. Wu,W.S., Jiang,Y.X., Chang,J.W. *et al.* (2018) HRPDviewer: human ribosome profiling data viewer. *Database (Oxford)*, **2018**, bay074.

6. Guo,H., Ingolia,N.T., Weissman,J.S. *et al.* (2010) Mammalian microRNAs predominantly act to decrease target mRNA levels. *Nature*, **466(7308)**, 835-840.
